# Equatorial pliosaurid from Venezuela marks the youngest South American occurrence of the clade

**DOI:** 10.1038/s41598-021-94515-8

**Published:** 2021-07-29

**Authors:** Dylan Bastiaans, Daniel Madzia, Jorge D. Carrillo-Briceño, Sven Sachs

**Affiliations:** 1 Palaeontological Institute and Museum, University of Zürich, Karl-Schmid-Strasse 4, 8006 Zürich, Switzerland; 2 Institute of Paleobiology, Polish Academy of Sciences, Twarda 51/55, 00-818 Warszawa, Poland; 3 Abteilung Geowissenschaften, Naturkunde-Museum Bielefeld, Adenauerplatz 2, 33602 Bielefeld, Germany

**Keywords:** Palaeontology, Taxonomy, Biodiversity, Stratigraphy

## Abstract

Pliosaurids were the dominant macropredators in aquatic environments at least since the Middle Jurassic until their extinction in the early Late Cretaceous. Until very recently, the Cretaceous record of Pliosauridae has been poor and difficult to interpret from the taxonomic and phylogenetic perspective. Despite that the knowledge of Cretaceous pliosaurids improved in recent years, numerous aspects of their evolutionary history still remain only poorly known. Here, we report the first pliosaurid material from Venezuela. The taxon is most likely earliest Cenomanian in age, thus representing the youngest occurrence of Pliosauridae from South America. The Venezuelan taxon is based on a well-preserved tooth crown whose morphology and outer enamel structural elements appear to resemble especially those observable in the giant pliosaurid *Sachicasaurus vitae* from the Lower Cretaceous of Colombia. The new discovery extends the pliosaurid record on the continent by more than 10 million years and likely marks the southernmost Upper Cretaceous occurrence of Pliosauridae, worldwide. We also briefly discuss the affinities of the enigmatic Venezuelan elasmosaurid *Alzadasaurus tropicus* and highlight similarities to elasmosaurids from the Western Interior Seaway*.*

## Introduction

With the appearance of the Thalassophonea, possibly around the Early-Middle Jurassic transitional interval^1^, pliosaurids became the dominant macropredators in the aquatic environments. They played an important role as apex predators in marine ecosystems until their extinction possibly around the middle Turonian (early Late Cretaceous) (e.g.,^1–6^). While Jurassic thalassophoneans have been intensively studied, especially those from the fossil-rich provenances in Europe (e.g.,^2,7–11^), the Cretaceous representatives have long been neglected; and were based on patchy occurrences (e.g.,^12–22^) with largely unexplored phylogenetic affinities (see, e.g.,^23^). Recent studies, including establishments of new taxa from the Lower Cretaceous of Colombia^24–26^ and Russia^27,28^ and from the Upper Cretaceous of the United States^3^, reports of newly discovered, isolated pliosaurid material^5,29–33^ as well as reassessment of historical material^4,31,34^, led to a better understanding of pliosaurid phylogeny (e.g.,^1,2,6,26–28,35,36^) and enabled some initial inferences of the divergence times and rates of their Cretaceous lineages^1^.

Despite that the knowledge of the Cretaceous thalassophonean pliosaurids has improved in recent years, still considerable efforts are necessary to obtain an accurate perception of the clade’s diversity, disparity, and dispersal patterns during the final tens of millions of years of their evolutionary history.

**Figure 1:**
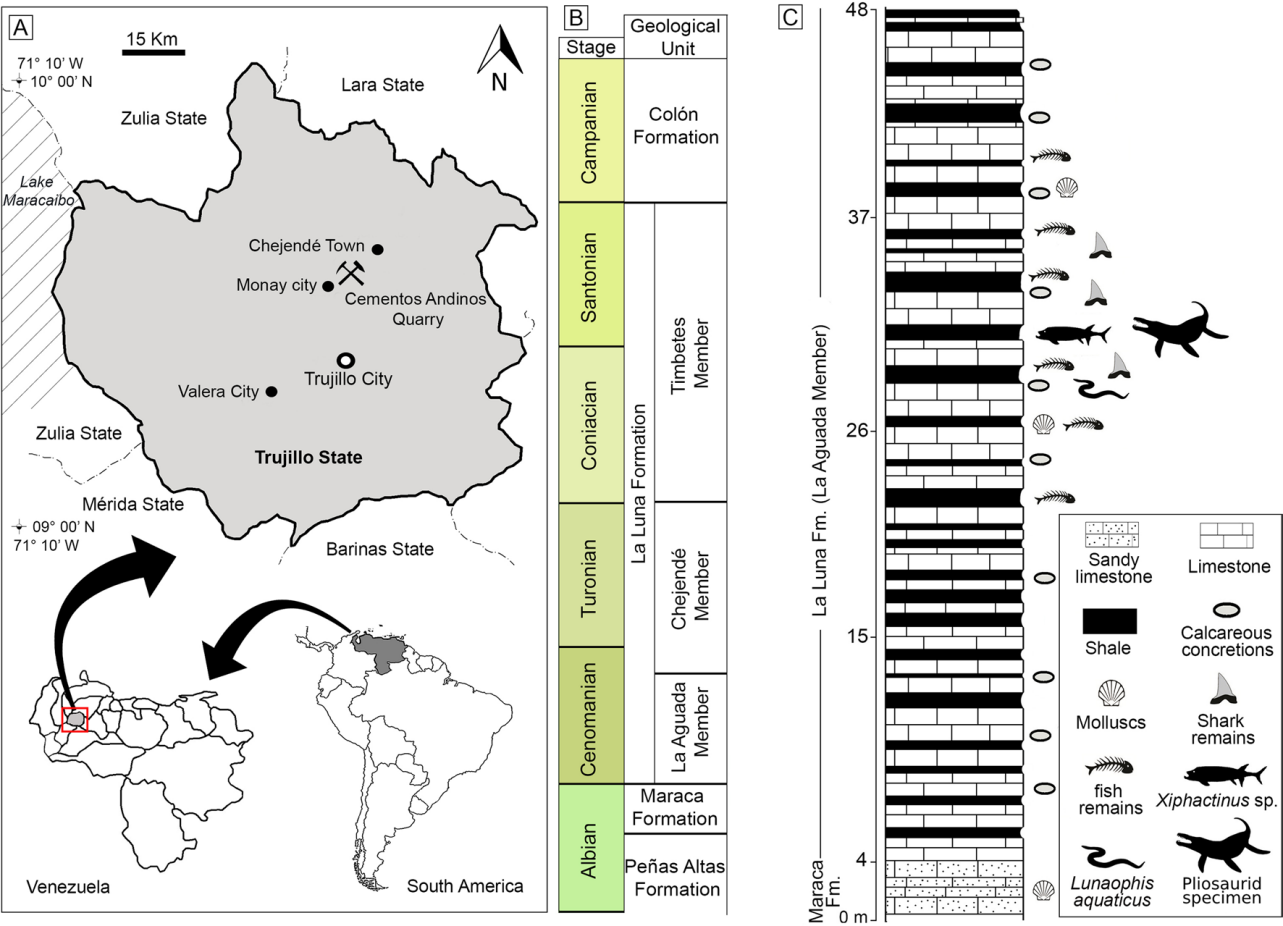
Location and geology of the “Cementos Andinos” quarry, Trujillo state, western Venezuela. (**A**) Location map. (**B**) Cretaceous lithostratigraphic units of the Chejendé region, near Monay city, Trujillo State (modified after^38,39^). (**C**) Stratigraphic section of the uppermost Maraca Formation and the La Aguada Member (La Luna Formation) in the “Cementos Andinos” quarry (modified by Jorge D. Carrillo‑Briceño after^37,40^ and using Adobe Illustrator & Photoshop [v2021.25.0]).

Here, we report the first pliosaurid material from Venezuela. The new specimen originates from the mid-Cretaceous succession of the La Luna Formation, Candelaria Municipality, Trujillo state, western Venezuela (Fig. 1)^37–40^. This find is significant because it represents the youngest record of Pliosauridae from the South American continent; younger by more than 10 million years than the late Aptian (late Early Cretaceous) ‘*Kronosaurus*’ *boyacensis*, the second youngest South American record^15^. It is also very likely the southernmost Upper Cretaceous occurrence of the clade, worldwide. The pliosaurid specimen also marks only the second plesiosaur record known from Venezuela. A partial elasmosaurid skeleton from the eastern part of the country (the affinities of which are discussed below) has been described in 1949 by Colbert^41^ and named *Alzadasaurus tropicus*.

**Figure 2:**
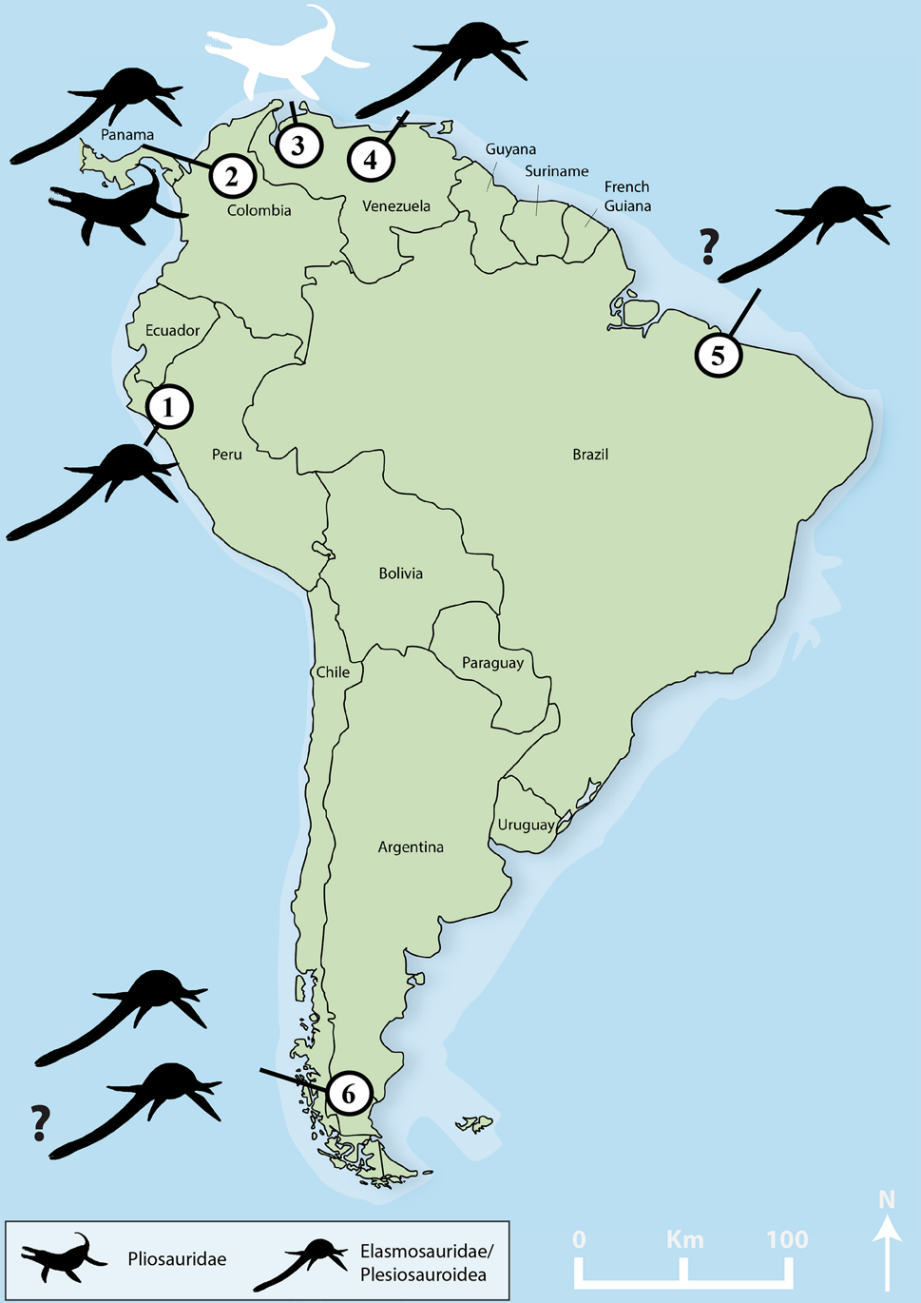
Distribution of mid-Cretaceous (Aptian-Turonian) plesiosaurs in South America. The new pliosaurid from the La Luna Formation is highlighted. (**1**) Jaén area, Romirón Fm., Peru; (**2**) Villa de Leiva, Paja Fm., Colombia; (**3**) Cementos Andinos Quarry, La Luna Fm., Venezuela; (**4**) Altagracia de Orituco area, Querecual Limestone, Venezuela; (**5**) Near Sao Luis, Alcântara Fm., Brazil; (**6**) Santa Cruz Province, Mata Amarilla Fm., Argentina. See Table 1 for details and references (map provided with permission by PD Dr. Torsten M. Scheyer; modified by Dylan Bastiaans using Adobe Illustrator & Photoshop [v2021.25.0]).

The Venezuelan pliosaurid material described herein is a valuable addition to the scarce plesiosaur record of the mid-Cretaceous—i.e., Aptian–Albian to Cenomanian–Turonian^42^—of South America (Fig. 2). Plesiosaurs, in general, are extraordinarily rare in the mid-Cretaceous of South America. They are often represented by isolated fragmentary material, with the notable exception of the taxa originating from the upper Aptian (Lower Cretaceous) of the Paja Formation in Colombia^15,36,43^, and are not diagnostic beyond larger clades (Table 1). Despite that the new material is represented by an isolated tooth crown, its excellent preservation allows for a detailed description of its morphology and the outer enamel structural elements. Through comparisons with other Cretaceous pliosaurids, and by using multivariate analyses of pliosaurid dental features that have recently become available^5^, it is possible to determine its taxonomic affinities and an approximate phylogenetic placement.

**Table 1:** Locality information and literature references on the mid-Cretaceous plesiosaurs of South America.

Original taxonomic assignment	Locality	Country	Unit	Stage^a^	Material	Current taxonomic assignment	LIC^b^
*Alzadasaurus colombiensis*^14^	Villa de Leiva	Colombia	Paja Formation	uA	Several skeletons and skeletal remains	*Callawayasaurus colombiensis*^36,43–47^	Elasmosauridae
*Leivanectes bernardoi*^36,43^	Villa de Leiva	Colombia	Paja Formation	uA	Anterior half of the skull	As original	Elasmosauridae
*Kronosaurus boyacensis*^15^	Villa de Leiva	Colombia	Paja Formation	uA	Largely complete skeleton	‘*Kronosaurus*’* boyacensis*^26^	Brachaucheninae
Pliosauroidea?/Pliosauridae? indet.^48^	Jaén area	Peru	Romirón Formation	uC–lT	Two vertebrae	Plesiosauria indet.	Plesiosauria
Elasmosauridae indet.^49^	Jaén area	Peru	Romirón Formation	uC–lT	5 cervical vertebrae	As original	Elasmosauridae
Plesiosauria indet.^50^	Near Sao Luís	Brazil	Alcântara Formation	C	Teeth	As original^51^	Plesiosauria
*Alzadasaurus tropicus*^41^	Near Altagracia de Orituco	Venezuela	Querecual limestone	C–T	Partial postcranial skeleton	Elasmosauridae indet.^44^^,c^	Elasmosauridae
Elasmosauridae indet.^52^	Near Tres Lagos	Argentina	Mata Amarilla Formation	C–S	Teeth, vertebrae, propodial	As original	Elasmosauridae
*Polyptychodon patagonicus*^53^	Santa Cruz Province	Argentina	?Mata Amarilla Formation	?C–S	Teeth	Plesiosauria indet.^52^	Plesiosauria

Welles^14,44^, Hampe^15^, Páramo-Fonseca et al.^26,36,43^, Colbert^41^, Carpenter^45^, Jaillard et al.^49^, Carvalho et al.^50^, O’Gorman and Varela^52^, Ameghino^53^, Jaimes and Parra^46^, Goñi and Gasparini^47^, Bôas and Carvalho^51^, Meza-Velez and O’Gorman^48^.

Note that Pliosauroidea?/Pliosauridae? indet. of^48^ may represent the same material as that of^49^.

^a^*A* Aptian, *C* Cenomanian, *T* Turonian, *l* lower, *u* upper.

^b^Least inclusive clade.

^c^This study.

### Institutional abbreviations

AMNH, American Museum of Natural History, New York, USA; CAMSM, Sedgwick Museum of Earth Sciences, University of Cambridge, Cambridge, UK; DMNS, Denver Museum of Nature and Science, Denver, USA; FMNH, Field Museum of Natural History, Chicago, USA; GFMSU, Geological Faculty of Lomonosov Moscow State University, Museum at the academic base named after Prof. A. A. Bogdanov, Bakhchisaray district, Crimea; MCNC, Museo de Ciencias Naturales de Caracas, Caracas, Venezuela; MWGUW, Stanislaw Józef Thugutt Geological Museum, Warsaw, Poland; UNSM, University of Nebraska State Museum of Natural History, Lincoln, USA.

### Geological and stratigraphic setting

**Figure 3:**
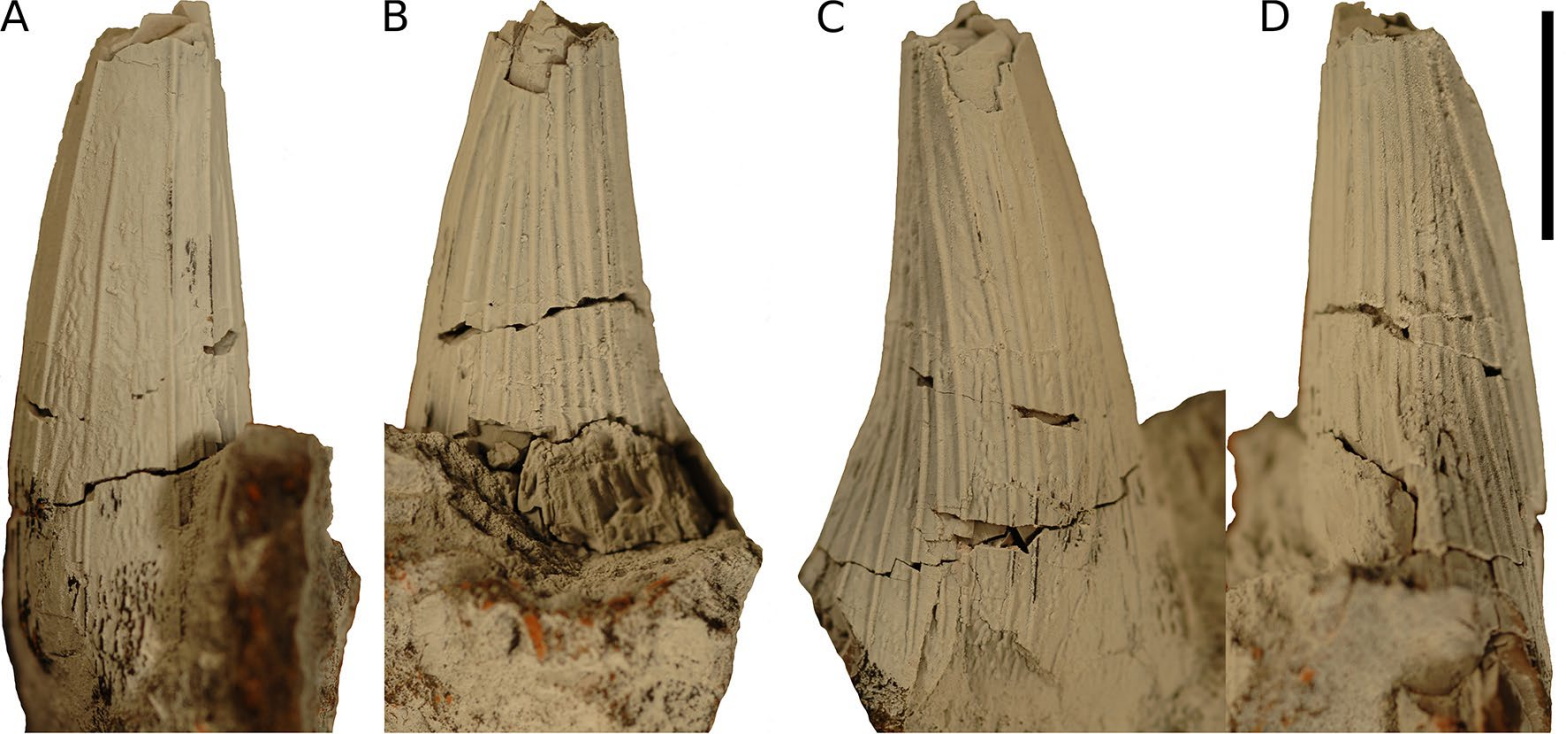
MCNC-1830 in (**A**) mesial, (**B**) lingual, (**C**) labial, and (**D**) distal view. Scale bar = 10 mm. Photographs taken by Dylan Bastiaans. Figure created by Daniel Madzia using the free online application Photopea (https://www.photopea.com/).

The specimen MCNC-1830 originates from the La Aguada Member of the La Luna Formation at the “Cementos Andinos” quarry, where calcareous rocks are mined for cement production. The quarry is located in the Andes range (Cordillera de Mérida), east of Lake Maracaibo, 10 km to the northeast of Monay city, Candelaria Municipality, Trujillo state, western Venezuela (Fig. 1A). MCNC-1830 was collected in situ in 2014 by one of the authors (JDCB), at the top of the quarry (9° 36′ 52″ N, 70° 24′ 3″ W), in the same outcrop of the La Aguada Member shown by^37^ (Fig. 3A in^37^) (Fig. 1).

The La Luna Formation is the most prolific petroleum source rock in western Venezuela and part of eastern Colombia^54–57^, and represents a marine sequence deposited under anoxic–dysoxic conditions along the passive margin of northern South America during the Cenomanian–Campanian^57^. The La Luna Formation is an extensive geological unit that spans the foreland of the southern Caribbean Ridge, including a large part of northwest of Venezuela (Sierra de Perijá to the Mérida Andes) and to Colombia, and gradually transitions east into the contemporaneous Rio Querecual Formation (eastern Venezuela) which is equivalent in facies^55^. These concretions range from a few centimeters to well over a meter in length (e.g.,^37^, Fig. 3C,D). In the southeast of the Maracaibo basin in the Lara and Trujillo states, the La Luna Formation is divided into three members (Fig. 1B): the La Aguada Member (bottom); the Chejendé Member (middle) and the Timbetes Member (top) (for a detailed description see^37,39,56^ and references therein). The thickness of the La Luna Formation ranges from 100 to 300 m, generally increasing northwards (^56,58^ and references therein).

In the Lara and Trujillo states, the La Aguada Member reaches a thickness of ~ 60 m^39^. The outcrops of the La Aguada Member, as exposed at the top of the “Cementos Andinos” quarry, consist of dense dark-grey limestones (less than ~ 60–70 cm thick), intercalated by compact and laminated black/dark-grey shales, and abundant calcareous concretions. MCNC-1830 derives from a black shale horizon that has produced ichnofossils, molluscs, chondrichthyans^40^, abundant osteichthyans^59^, and a marine snake^37,60^. The base of the La Aguada Member at the “Cementos Andinos” quarry overlays a fossiliferous dark-grey sandy limestone (personal observation, Fig. 1C) that has been identified as the top of the upper Albian Maraca Formation in the Andes of Trujillo and Lara states^38^. Other authors (e.g.,^61,62^) have used the term the ‘La Puya member’ to refer to a thin section (< 30 m) at the top of the Peñas Altas Formation in the Andes of Lara and Trujillo (Fig. 1). Therefore, the discrepancy between the use of the Maraca Formation or the ‘La Puya Member’ for the thin sequence under the La Aguada Member is still unresolved^37^.

The precise age of the La Aguada Member and its corresponding sections across Venezuela and Colombia remains uncertain, ranging from Albian-Cenomanian^55,61,63–65^, lower–upper Cenomanian^39,63–69^ and even Cenomanian-Santonian^70,71^. Most relevant, perhaps, is the dating of the La Peña/San Felipe Sections by^72^, located in the eastern part of the Maracaibo Basin on the eastern edge of the village of Chejendé, Trujillo, which is less than 10 km from the “Cementos Andinos” quarry. Based on nannofossils, the La Aguada Member of Chejendé was deposited no earlier than in the latest Albian to middle Cenomanian interval^72^. However, only the base of the La Aguada Member was exposed, thus strongly suggesting an earliest Cenomanian age for MCNC-1830 that was recovered much higher in the section (^72^, p. 352 and Fig. 3A). Despite the debate on the exact age of the base of the La Luna Formation east of Lake Maracaibo (e.g., La Aguada Member), it seems that, based on the ammonite record, it becomes progressively younger westward^63–65^. For a detailed discussion on the age of the La Aguada Member see Supplementary Information 1.

### Paleoenvironment and other vertebrates

During much of the Early Cretaceous, what is today Venezuela was covered by an epicontinental sea that rapidly transgressed during the latest Albian and Cenomanian towards the craton followed by a period of re-oxygenation^55^. The La Aguada Member has often been considered to cover a transitional environment between the shallow water conditions of the Maraca Formation (La Puya Member) and the pelagic low energy conditions of the La Luna Formation; however, water depths never exceeded 50 m^38,40,55,73^. The La Aguada Member has intervals rich in organic matter which have been suggested to be of algal origin^40^. Sedimentological and invertebrate (micro)fossil proxies indicate a shallow water environment (presence and abundance of globigerinid and a scarcity of globotruncanid foraminifera) with oxygenated and generally nutrient-rich surface waters and a stratified water column seem to have been present^40,55^. The La Luna Formation is associated with an outer shelf/upper slope paleoenvironment with a high diversity of medium to large marine vertebrates (see Supplementary Table 1) that would have served as ample food resources for opportunistic predators^55^. Nonetheless, the vertebrate record of the La Aguada Member remains fairly limited. A wide variety of bony fish remains have been uncovered, including scales, isolated and semi-articulated cranial and postcranial remains of *Xiphactinus*^59^, other ichthyodectiforms, enchodontids, and small indeterminate fishes^40,64^. A high diversity of lamniform sharks (at least 12 taxa in five clades, eight of which are anacoracids), have been described from the La Luna Formation, representing active pelagic predators and scavengers of large vertebrates and small nektobenthic predators feeding on small bony fish and invertebrates^40^. The new plesiosaur specimen adds to the diversity of large marine reptiles from the La Luna Formation and represents the largest predator described from the strata so far (^74,75^, Supplementary Table 1).

Bottom water conditions were predominantly anoxic or suboxic, as indicated by the scarcity of benthic invertebrates with only rare occurrences of small bivalve moulds in the limestones (and undetermined ammonites), and some inoceramids in the calcareous concretions and a lack of reworking by bioturbation and/or high-water energy conditions (for more details see the Supplementary Information 1;^40,55^). It seems that anoxic sedimentation, possibly related to upwelling along the northwestern coast of South America, was widespread across the Venezuelan and Colombian platform and possibly even spanning a major part of northern South America and the southern Caribbean during the Cenomanian–Santonian^55^. Towards the top of the Chejendé Member oxygen and nutrient conditions improve and pelecypods and ammonites are more frequent in the concretion-rich portion of the section^55^.

## Material and methods

### Material

The study is based on an isolated tooth crown belonging to a brachauchenine pliosaurid of probable early Cenomanian (early Late Cretaceous) age. The specimen originates from the La Aguada Member of the La Luna Formation, Candelaria Municipality, Trujillo state, western Venezuela. It is housed at the Museo de Ciencias Naturales de Caracas in Caracas, Venezuela (MCNC) under the catalog number MCNC-1830 (Fig. 3).

The specimen (MCNC-1830) was found as part of a larger collection of fossil vertebrates at the “Cementos Andinos” quarry with the support of the authorities of the mining company. Legal authorization was issued by the Instituto del Patrimonio Cultural de Venezuela (IPC) through the collection permit No. 000327/2013, and through permission for mobilization and study No. 071/2015.

### Multivariate analyses

To further assess the taxonomic affinities of MCNC-1830 and to explore its morphospace occupation among thalassophonean pliosaurids, we performed cluster and principal coordinates analyses using the dataset of^5^. This dataset was constructed to collectively summarize the dental morphological features present in Thalassophonea. The Venezuelan taxon (MCNC-1830) and *Sachicasaurus vitae*^26^ from the upper Barremian of Sáchica, Colombia, were added to this dataset. The former was scored based on personal observations and the latter based on^26^ and following discussions with Cristian David Benavides-Cabra (Universidad Nacional de Colombia, Bogotá, Colombia). We further modified the carinal score (character 3) for GFMSU h-216 (0 → 2), hereafter termed the ‘Crimean pliosaurid’, that was miss-scored in the original version of the dataset as lacking the carinae, while, in fact, the crown has two carinae^5,29^. See Supplementary Information 1 for the matrix.

We replicated the protocol of^5^; we applied a 50% completeness threshold to remove the influence of taxa based on insufficiently complete/preserved material, scaled the data to equal variance and a mean of zero through subtraction of the mean value for each character and then divided it by the standard deviation. A distance matrix was created using the Gower metric, that is well suited for datasets that comprise both continuous and discrete variables^76^. We used the cluster v2.1.0 package in the R statistical environment (RStudio Version 1.2.5033^77^); from the resulting distance matrix a cluster dendrogram analysis using the stats base package and the Ward.D2 method was produced.

The same matrix was used to explore the dental morphospace occupation of particular thalassophonean taxa through a principal coordinates analysis, using ape v5.3^78^. We again used the Gower metric and applied the Cailliez correction for negative eigenvalues. See Supplementary Information 2 for the R code.

### The terminology of tooth crown orientation and morphology

We follow the crown orientation terminology of^79^: apical, toward the crown apex; basal, toward the *cervix dentis*; distal, away from the tip of the snout; labial, toward the lips; lingual, toward the tongue; mesial, toward the tip of the snout. The morphological traits exposed on the outer enamel surface are described using the nomenclature as adopted by^5,6^: apicobasal ridges, longitudinally running enamel ridges of variable apicobasal extent that can be developed around the entire crown circumference and are approximately semicircular or triangular in cross-section; ridglets, subtle apicobasally-expressed enamel structures that are often developed between adjacent apicobasal ridges or on an unridged enamel surface; the ridglets may be very indistinct as well as produce a distinct vermicular pattern (see^4^: Fig. 7).

### Systematic paleontology

Plesiosauria^80^

Pliosauridae^81^

Thalassophonea^35^

Brachaucheninae^35^

Brachaucheninae indet.

Figure 3

#### Material

MCNC-1830, an isolated tooth crown (height of the preserved part =  ~ 30 mm).

#### Occurrence

(Most likely) lower Cenomanian, Upper Cretaceous from the La Aguada Member, La Luna Formation (see ‘Geological and stratigraphic setting’ for detailed information with respect to the stratigraphic context).

#### Description and comparisons

The apicalmost part of MCNC-1830 is broken off and the basal section is slightly compressed in labiolingual direction. Linguodistally, part of the crown is crushed near the base. The crown is conical (subcircular in cross-section), as in *Acostasaurus pavachoquensis*^25^, *Brachauchenius lucasi*^20,22^, ‘*Kronosaurus*’ *boyacensis*^15^, *Kronosaurus queenslandicus*^82^, *Megacephalosaurus eulerti*^6^, ‘*Polyptychodon*’ *hudsoni* (DM, pers. obs.), *Sachicasaurus vitae*^26^, and the element-rich assemblage collectively assigned to ‘*Polyptychodon interruptus*’ reappraised by^4^ as probably belonging to multiple taxa, but differing from *Luskhan itilensis*^28^, *Makhaira rossica*^27^, *Stenorhynchosaurus munozi*^24^, and the ‘Crimean pliosaurid’^29^ that possess trihedral (*M. rossica*), subtrihedral (*L. itilensis*, *S. munozi*), and trihedral-to-‘trapezoid’ (the ‘Crimean pliosaurid’) cross-sectional shapes of their tooth crowns. No carinae/cutting edges are present, unlike the condition observable in *L. itilensis*, *M. rossica*, *S. munozi*, and the ‘Crimean pliosaurid’, which are characterized by the presence of one (*L. itilensis*, *S. munozi*), two (the ‘Crimean pliosaurid’), and three (*M. rossica*) carinae. The apicobasal ridges in MCNC-1830 are approximately semicircular in cross-section and are developed around the entire circumference though they are most densely packed linguodistally. All of the ridges appear to reach the base of the crown, as is widespread among brachauchenines^5^. Some are very pronounced and likely reached the apex though due to the lack of the apical part, this cannot be confirmed. Some of the ridges are approaching each other on the linguodistal part of the crown, around the mid-section, but no ridges have been observed to branch, unlike in *Brachauchenius lucasi*, ‘*Polyptychodon*’ *hudsoni*, and *Megacephalosaurus eulerti* that typically show clear branching ridges around the mid-sections of tooth crowns. Mesiolabially, the enamel surface exposed between the apicobasal ridges shows well-pronounced ridglets, forming a vermicular pattern, similar to the state observable in *Sachicasaurus vitae* and some specimens from the ‘*Polyptychodon*’ assemblage, such as CAMSM B 75754.

#### Assessment through multivariate analyses

**Figure 4:**
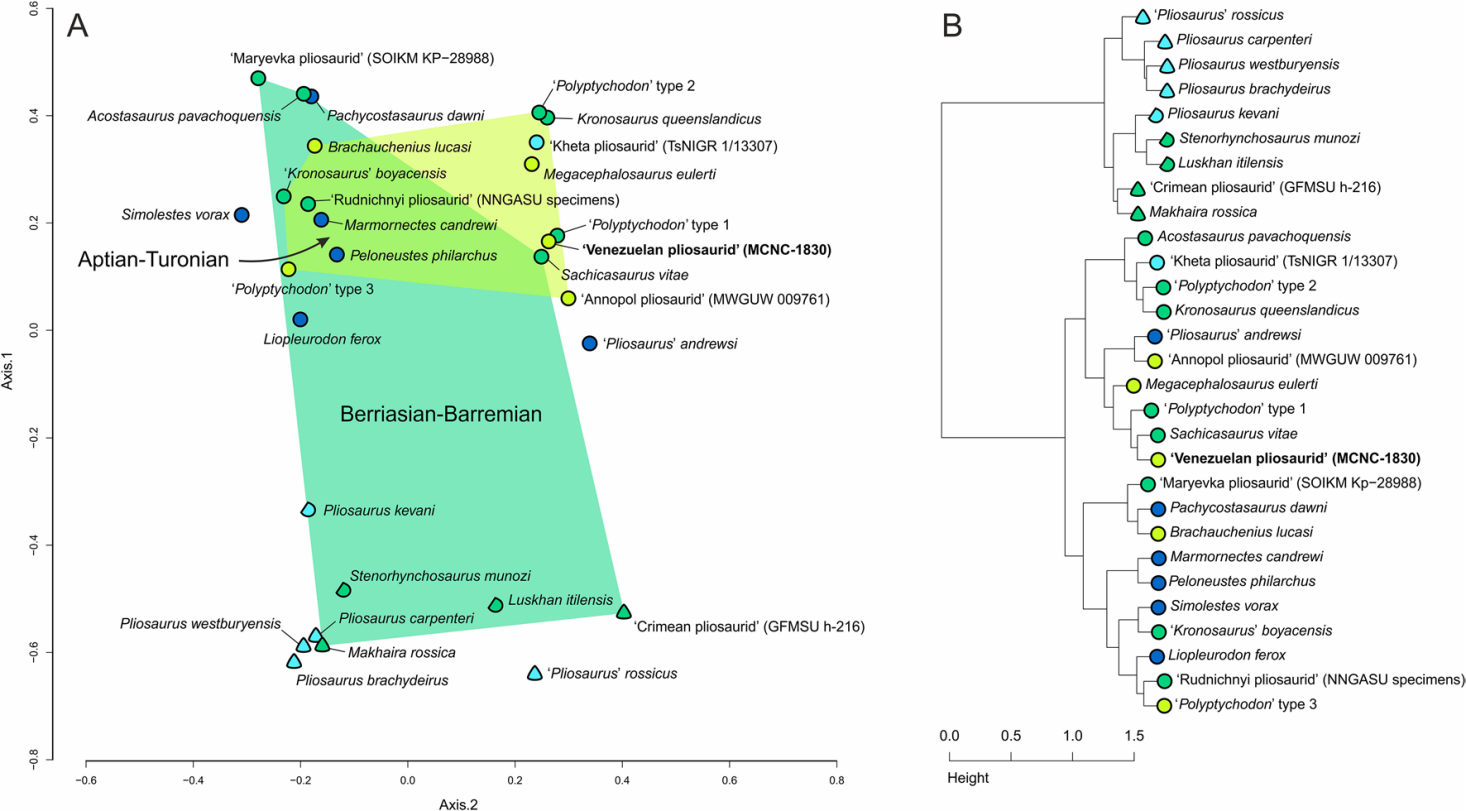
Results of the principal coordinates analysis (**A**), showing the morphospace occupation of MCNC-1830 among Jurassic and Cretaceous pliosaurids, using principal coordinates 1 and 2, and results of the cluster analysis (**B**). Graphic representation and color scheme of the results follow^5^: circles—crowns with subcircular cross-section; semicircles—crowns with subtriangular cross-section; triangles—crowns with approximately triangular cross-sections. Graphic results exported from RStudio Version 1.2.5033^77^; figure created by Daniel Madzia using CorelDraw X8 (v18.1.0.661).

The results of our multivariate analyses are broadly similar to those of^5^. The principal coordinates analysis (PCoA) as well as the cluster analysis recognize the presence of two general tooth crown ‘morphogroups’ in pliosaurids, one comprising the crowns with a conical shape (subcircular cross-section) and the other one including those with the trihedral/subtrihedral morphology (triangular/subtriangular cross-section) (Fig. 4). As in^5^, PCoA largely separates the two ‘morphogroups’ by the first principal coordinate axis (Fig. 4A). The specimen MCNC-1830 is placed on the positive sides of the first and second axis, in close proximity to *Sachicasaurus vitae* and the ‘*Polyptychodon*’ type 1, a tooth morphotype from the middle to upper Albian Gault Formation and the lowermost Cenomanian Cambridge Greensand Member of the West Melbury Marly Chalk Formation (late Albian in age), represented by a number of tooth crowns of differing sizes that are characterized especially by their arrangements of apicobasal ridges (^4^: Figs. 3A, 4A, and 5). These crowns differ from MCNC-1830 in that their enamel surface is smoother than in MCNC-1830 and do not include well-pronounced ridglets.

A similar result, to that of the PCoA, was obtained through the cluster analysis that placed MCNC-1830 in a cluster with *S. vitae* and further with ‘*Polyptychodon*’ type 1 and *Megacephalosaurus eulerti* (Fig. 4B), within the ‘conical’ part of the cluster dendrogram.

## Discussion

### Dental disparity of the Cretaceous pliosaurids

The results of our multivariate analyses differ in some aspects from those of^5^, which warrants some discussion. The addition of the late Barremian brachauchenine *Sachicasaurus vitae* expands the crown morphospace occupation of the pre-Aptian taxa towards the positive side of the second coordinate axis, further supporting the hypothesis that the latest brachauchenines experienced a substantial decrease in their dental disparity.

The most significant difference is the placement of GFMSU h-216 (‘Crimean pliosaurid’) within the ‘trihedral’ cluster. The analysis of^5^ placed the specimen among the conical-toothed taxa, in a cluster together with the Callovian (Middle Jurassic) taxon ‘*Pliosaurus*’ *andrewsi* and the Cenomanian (Late Cretaceous) specimen MWGUW 009761 (‘Annopol pliosaurid’), none of which is particularly reminiscent of the trihedral-to-‘trapezoid’ morphology of GFMSU h-216. It is worth noting that MWGUW 009761 shows a cross-section somewhat resembling a triangular shape, and may thus be characterized as being gently subtrihedral^31^. However, considering its overall morphology and its enamel character state distribution, we did not alter any scores for this specimen in the current version of the dataset.

The difference in the placement of GFMSU h-216 between^5^ and our study clearly stems for the correction of the carinal score for the specimen (0 → 2). ‘*Pliosaurus*’ *andrewsi* and MWGUW 009761 still cluster together among the conical-toothed pliosaurids.

### Remarks on the Venezualan elasmosaurid *Alzadasaurus tropicus*

The most complete plesiosaur find from Venezuela is a partial postcranial skeleton (AMNH 6796) that was discovered by a Venezuelan oil company near the vicinity of Altagracia de Orituco, eastern Venezuela and established as *Alzadasaurus tropicus* by Colbert^41^. Preserved are the posteriormost cervical vertebra, four pectoral and eight dorsal vertebrae, parts of associate ribs, the left scapula, a nearly complete left and parts of the right coracoid, a left humerus as well as parts of the left radius, ulna and carpus (^41^, p. 4). *A. tropicus* was considered a ‘*nomen vanum*’ by^44^ (p. 54), a taxon that was adequately described but lacks sufficient diagnostic characters (*nomen dubium* of current use). Following Colbert’s (^41^, p. 5) diagnosis for *A. tropicus*, the taxon is characterized by vertebrae with round centra and rather high, compressed, neural spines; scapula that has a broad dorsal process and a fairly broad ventral plate, not contributing to a median midline bar; an elongated coracoid that is expanded along the posterior margin, having a long posterior coracoid blade; coracoids which meet along a median symphysis anteriorly, being separated by an elongated median vacuity posteriorly; and humerus that is elongated and distally expanded.

Similar posterior cervical, pectoral or dorsal vertebrae with roundish centra and high, transversely compressed neural spines are present in various elasmosaurid taxa, including *Callawayasaurus colombiensis* (^44^, Plate 3, Fig. C), *Thalassomedon haningtoni* (S. Sachs pers. obs. November 2015), *Futabasaurus suzukii* (^83^, Fig. 5H), or *Elasmosaurus platyurus* (^84^, Fig. 5). A scapula with a broad dorsal process and an anteriorly broad ventral plate, lacking a pectoral bar, is a condition reminiscent of *T. haningtoni* (see^85^, Fig. 14). Transversely expanded anterior scapulae are present, e.g., in *Libonectes morgani* (^86^, Fig. 2) or *Elasmosaurus platyurus* (^44^, Fig. 14) where the scapulae form a posteromedial link to the anteromedial coracoids, called the pectoral bar. The coracoid of *A. tropicus* is expanded along the posterior margin, having a long posterior coracoid blade. The post-symphyseal coracoid, also called the coracoid blade, is usually shorter relative to the complete length of the coracoid and wider posteriorly (see e.g.,^41^, Fig. 8,^45^, Fig. 6 for comparison). A similar elongate and narrow coracoid blade is present in the latest Cretaceous elasmosaurids *Hydrotherosaurus alexandrae* where the coracoid blade is, however, less elongate relative to the complete length (see^85^, Fig. 8,^87^, Fig. 3), and *Aphrosaurus furlongi* where the posterior parts of the coracoid blades are pronouncedly transversely expanded (^85^, Fig. 23,^88^, Fig. 6G). The Turonian *Libonectes morgani* has clearly shorter coracoid blades (^86^, Fig. 2). The condition in the potentially coeval Cenomanian *Thalassomedon haningtoni* is unknown as the post-symphyseal coracoids of the holotype specimen (DMNS 1588) are not preserved. The type specimen of *Alzadasaurus* (FMNH 12009), described as *Al. riggsi* by^85^, was assigned to *Thalassomedon* by^45^. However, this specimen is an immature individual and most of the remains are fragmentary, heavily distorted and therefore insufficient for a confident diagnosis. The material, currently under study by SS & DM, therefore cannot be unambiguously assigned to *T. haningtoni* and is best considered a *nomen dubium*, following^89^. The coracoids typically connect anteriorly along their medial symphysis, and also the posterior intercoracoid vacuity is a condition characteristic for elasmosaurid plesiosaurs (see discussion in^90^). Humeri that are elongated and distally expanded are likewise found in several elasmosaurid taxa, such as *Thalassomedon haningtoni* (^85^, Fig. 15), *Libonectes morgani* (^91^, Fig. 7), *Futabasaurus suzukii* (^83^, Fig. 8A,B) or *Aphrosaurus furlongi* (^88^, Fig. 8).

In summary, all characters considered diagnostic for *Alzadasaurus tropicus*, as discussed by^41^, are also present in the potentially coeval Cenomanian elasmosaurid *Thalassomedon haningtoni*, known from the midwestern USA (see discussion in^92^). Only the shape of the coracoid blade cannot be confirmed as the post-symphyseal coracoid parts are missing in the *Thalassomedon* holotype specimen (DMNS 1588) and the pectoral girdles are not preserved in the referred specimen (UNSM 50132) either. Several characters considered diagnostic for *A. tropicus* by^41^ are typical for elasmosaurids in general, such as the intercoracoid vacuity.

Even though we have not observed any characters that could be used to diagnose *Alzadasaurus tropicus*, confirming its status as a *nomen dubium* or its similarity to *Thalassomedon haningtoni*, which would be suggested based solely upon Colbert’s^41^ publication, is beyond the scope of this study.

### Other potential pliosaurid specimens from the Upper Cretaceous of South America

It is worth noting that two partly damaged isolated vertebral centra from the Cenomanian Romirón Formation of Peru have been recently mentioned as possibly representing a pliosaurid (referred by Meza-Velez and O’Gorman^48^ to as ‘Pliosauroidea? indet.’ and ‘Pliosauridae? indet.’). However, the description of these incomplete specimens, representing a caudal and a sacral or proximal caudal centrum, has not been supplemented with detailed comparisons of contemporary taxa. A morphometric analysis of the specimens placed them outside the Elasmosauridae which led to the conclusion that pliosaurids are the most plausible group. However, similar proportions and a similar morphology (centra that are wider than long/high and higher than long, having amphicoelous articular faces) are also known for coeval early Late Cretaceous polycotylids (see e.g.^,93^). This group was not considered in the comparisons by Meza-Velez and O’Gorman^48^. For that reason, and owing to the apparent homoplasy in plesiosaur vertebral characters (e.g.,^94^), the taxonomic assignment is questionable. Pending more detailed assessment of the material and vertebral character distribution within Plesiosauria, the vertebrae are probably best interpreted as Plesiosauria indet.

## Conclusions

With the notable exception of the pliosaurid and elasmosaurid specimens originating from the upper Aptian (Lower Cretaceous) strata of the Paja Formation in Colombia, the plesiosaur record from the mid-Cretaceous (approximately encompassing the Aptian-Turonian interval) of South America is based on fragmentary remains of indeterminate phylogenetic affinities.

Here, we report the first pliosaurid material from Venezuela. The specimen originates from the La Aguada Member of the La Luna Formation in the Andes range (Cordillera de Mérida), east of Lake Maracaibo, 10 km to the northeast of Monay city, Candelaria Municipality, Trujillo state, western Venezuela. It was discovered in strata most likely deposited in the early Cenomanian (earliest Late Cretaceous). Despite comprising a single tooth crown, the preservation of the specimen allows for a detailed description, comparisons to teeth of other Cretaceous pliosaurids, and an assessment through multivariate analyses of data that have become available recently.

The overall morphology of the Venezuelan specimen and the distribution of its outer enamel structural elements indicate affinities to late-diverging brachauchenines and appear to resemble especially those observable in *Sachicasaurus vitae*, a recently described giant pliosaurid from the upper Barremian (Lower Cretaceous) of Boyacá, Colombia.

The most complete plesiosaur material from Venezuela described to date includes a partial postcranial specimen, established as the type of *Alzadasaurus tropicus*. The taxon is usually considered to lack diagnostic features and is treated as a *nomen dubium*. Our preliminary assessment of the specimen concurs with this though we have also observed characters that are shared with the middle Cenomanian (lower Upper Cretaceous) elasmosaurid *Thalassomedon haningtoni* from the midwestern USA.

In turn, the Venezuelan pliosaurid represents the youngest South American representative of the clade, over 10 Ma younger than the second youngest South American record (‘*Kronosaurus*’ *boyacensis*). Additionally, if the early Cenomanian age for the deposition of the fossil-bearing strata proves correct, the newly described specimen also marks the southernmost Upper Cretaceous occurrence of Pliosauridae, worldwide. Regardless, the Venezuelan pliosaurid represents a significant addition to the scarce record of the mid-Cretaceous plesiosaurs of South America and is another indicator of the potential and abundance of marine vertebrates from the Cretaceous of Venezuela.

## Supplementary Information

Supplementary Information 1.

Supplementary Information 2.

Supplementary Table 1.
