# Supplementary material for: Equatorial pliosaurid from Venezuela marks the youngest South American occurrence of the clade
Source: Sci Rep. 2021 Jul 29;11:15501. doi: 10.1038/s41598-021-94515-8 (PMC8322105; doi:10.1038/s41598-021-94515-8)
Supplement: Supplementary file 1 — Supplementary Information 1. [file 41598_2021_94515_MOESM1_ESM.docx]

**Equatorial pliosaurid from Venezuela marks the youngest South American occurrence of the clade**

**Supplementary Information 1**

Dylan Bastiaans^1^, Daniel Madzia^2*^, Jorge D. Carrillo-Briceño^1^ & Sven Sachs^3^

^1^Palaeontological Institute and Museum, University of Zürich, Karl-Schmid-Strasse 4, 8006 Zürich, Switzerland; ORCID: https://orcid.org/0000-0002-3096-2062; ORCID: https://orcid.org/0000-0002-8652-7692, respectively

^2^Institute of Paleobiology, Polish Academy of Sciences, Twarda 51/55, 00-818 Warszawa, Poland; ORCID: https://orcid.org/0000-0003-1228-3573

^3^Naturkunde-Museum Bielefeld, Abteilung Geowissenschaften, Adenauerplatz 2, 33602 Bielefeld, Germany

*Corresponding author: Daniel Madzia (daniel.madzia@gmail.com)

**STRUCTURE**

**Character matrix 2**

**Extended stratigraphic and paleoenvironmental information 3**

**Character matrix**

| TAXON | C1 | C2 | C3 | C4 | C5 | C6 | C7 | C8 | C9 | C10 | C11 |
| --- | --- | --- | --- | --- | --- | --- | --- | --- | --- | --- | --- |
| *Marmornectes candrewi* | 25.3 | 2.976470588 | 0 | 1 | 0 | 0 | 0 | 1 | 1 | 0 | 1 |
| *Pachycostasaurus dawni* | 33 | 1.736842105 | 0 | 1 | 1 | 0 | 0 | 0 | 1 | 0 | NA |
| ‘*Pliosaurus*’ *andrewsi* | 41.1 | 1.763948498 | 0 | 0 | 0 | 0 | 1 | 1 | 0 | 0 | 2 |
| *Peloneustes philarchus* | 31.3 | 2.576131687 | 0 | 1 | 0 | 0 | 0 | 1 | 0 | 0 | 1 |
| *Simolestes vorax* | 82.4 | 2.559006211 | 0 | 1 | 0 | 0 | NA | 0 | 1 | 2 | 1 |
| *Liopleurodon ferox* | 81 | 2.436090226 | 0 | 1 | 0 | 0 | NA | 1 | 0 | 1 | 2 |
| *Anguanax zignoi* | 18 | 1.5 | NA | NA | NA | 0 | 0 | 2 | NA | NA | NA |
| *Gallardosaurus itturraldei* | NA | NA | 1 | 0 | NA | 1 | 0 | NA | NA | NA | NA |
| *Pliosaurus brachydeirus* | NA | 2.2 | 2 | 1 | 0 | 2 | 1 | 2 | 0 | 1 | 0 |
| *Pliosaurus kevani* | 48 | 1.655172414 | 1 | 1 | 0 | 1 | 1 | NA | 0 | 1 | NA |
| *Pliosaurus westburyensis* | 110 | 2.2 | 2 | 1 | 0 | 2 | 1 | 2 | 0 | 1 | 0 |
| *Pliosaurus carpenteri* | 100 | 2 | 2 | 1 | 0 | 2 | 1 | 2 | 0 | 1 | 0 |
| ‘*Pliosaurus*’ *rossicus* | 130 | 3.25 | 2 | 0 | 0 | 2 | 1 | 2 | 0 | 1 | 0 |
| ‘Kheta pliosaurid’ | 63 | 1.96875 | 0 | 0 | 0 | 0 | 0 | 0 | 1 | 0 | 1 |
| ‘Maryevka pliosaurid’ | 28 | 1.866666667 | 0 | 1 | 1 | 0 | 0 | 0 | 2 | 1 | 1 |
| ‘Rudnichnyi pliosaurid’ | 75 | 1.88 | 0 | 1 | 0 | 0 | 0 | 0 | 0 | 1 | 2 |
| ‘Crimean pliosaurid’ | 22 | 1.466666667 | 2 | 0 | 0 | 2 | 1 | 2 | 0 | 0 | NA |
| *Makhaira rossica* | 48 | 3 | 3 | 1 | 0 | 2 | 1 | 2 | 0 | 0 | NA |
| *Luskhan itilensis* | NA | NA | 1 | NA | 0 | 1 | 1 | 2 | 0 | 0 | NA |
| *Acostasaurus pavachoquensis* | NA | NA | 0 | 1 | 0 | 0 | 0 | 0 | 2 | 0 | NA |
| *Stenorhynchosaurus munozi* | NA | NA | 1 | 1 | 0 | 1 | 1 | 2 | 0 | 0 | 0 |
| *Kronosaurus queenslandicus* | 100 | 2 | 0 | 0 | 0 | 0 | 0 | 0 | 2 | 0 | NA |
| ‘*Kronosaurus*’ *boyacensis* | 90 | 2.5 | 0 | 1 | 0 | 0 | 0 | 0 | 0 | NA | 1 |
| *Megacephalosaurus eulerti* | 89 | 1.854166667 | 0 | 0 | 1 | 0 | 0 | 0 | 0 | 0 | 0 |
| *Brachauchenius lucasi* | 25 | 2 | 0 | 1 | 1 | 0 | 0 | 0 | 0 | 0 | NA |
| ‘*Polyptychodon*’ type 1 | 70 | 2 | 0 | 0 | 0 | 0 | 0 | 1 | 0 | 0 | 1 |
| ‘*Polyptychodon*’ type 2 | 70 | 2 | 0 | 0 | 0 | 0 | 0 | 0 | 2 | 0 | 1 |
| ‘*Polyptychodon*’ type 3 | 95 | 1.9 | 0 | 1 | 0 | 0 | 0 | NA | 0 | 1 | NA |
| ‘Annopol pliosaurid’ | 36 | 1.8 | 0 | 0 | 0 | 0 | 1 | 0 | 0 | 0 | 1 |
| *Sachicasaurus vitae* | 90 | 1.285714286 | 0 | 0 | 0 | 0 | 0 | 1 | 0 | 1 | NA |
| ‘Venezuelan pliosaurid’ | 30 | 1.5 | 0 | 0 | 0 | 0 | 0 | NA | 0 | 1 | NA |

**Extended stratigraphic and paleoenvironmental information**


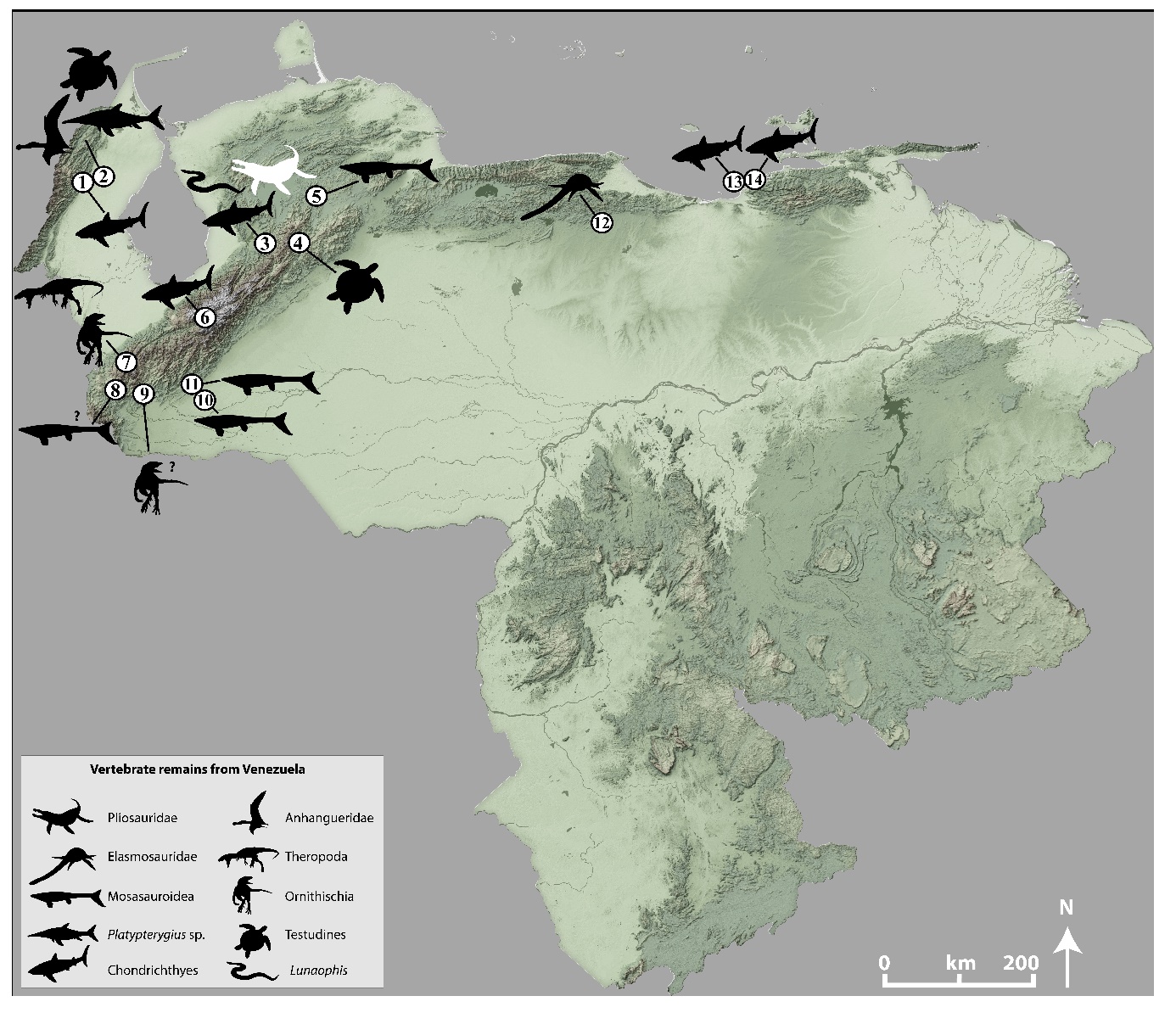


**Supplementary Figure 1.** Mesozoic vertebrates (silhouettes) and vertebrate-bearing localities (numbers in circles) of Venezuela.
(1) La Luna Quarry, Sierra de Perijá, Zulia State; (2) Rosarito Quarry, Sierra de Perijá, Zulia State; (3) Cementos Andinos Quarry, Candelaria Municipality, Trujillo State; (4) Humocaro Alto caves, Morán Municipality, Lara State; (5) “Bloques de Pozo Guapo”, Torres Municipality, Lara State; (6) Mucujún River Basin, Libertador Municipality, Mérida State; (7) La Quinta Formation, Jáuregui Municipality, Táchira State; (8) Táriba-Cordero, Cárdenas Municipality; (9) La Fundación, Fumento Municipality, Táchira State; (10) Santa Bárbara, Santa Bárbara Municipality, Monagas State; (11) unnamed location northwest of Santa Bárbara, Santa Bárbara Municipality, Monagas State; (12) Altagracia de Orituco, José Tadeo Monagas Municipality, Guárico State; (13) La Borracha Island, Anzoátegui State; (14) Chimana Grande Island, Anzoátegui State. Note that more localities exist but could not be assigned to a specific region. In addition, fish localities are not shown here but are mentioned in Supplementary Table 1 with all other vertebrate remains and their literature references. Modified by Dylan Bastiaans after [1, 2] and using Adobe Illustrator & Photoshop (v2021.25.0). The digital shaded relief map of Venezuela was created as a composite of 3-arc-second elevation data, captured during the Shuttle Radar Topography Mission (SRTM, February 2000) [1,2]).

**Precise age of the La Luna Formation**

Garner [3] ascribed the lithostratigraphic unit the name the ‘La Luna Limestone’ in the Quebrada La Luna of the Perijá range (Zulia state, western Venezuela) ([4]). Subsequently, Hedberg and Sass [5] formally described it as a formation. An alternation of black or dark-gray limestones and organic calcareous shales with abundant calcareous concretions characterize the general lithology of the La Luna Formation ([4, 6-8]).

The precise age of the La Aguada Member and its corresponding sections across Venezuela remains uncertain. The La Aguada Member in Trujillo and Lara State has been regarded to represent lower-middle Cenomanian ([9]), based on a combination of planktonic foraminifera and ammonites, as well as Albian-Cenomanian ([10-12]) based on ammonites only (e.g., *Mariella worthensis*, *Hamites* cf. *virgulatus*, *Anisoceras* *perarmatum* of the Barbacoas syncline, La Puya Mb./La Aguada Mb., southwest Lara State). Tribovillard et al. ([7], fig. 3) also seem to indicate a latest Albian-Cenomanian age for the deposition of the base of the La Aguada Member in the San Pedro and Trujillo section (east of Lake Maracaibo) based on ammonites (i.e., *Mortoniceras inflatum* zone) and based on foraminiferal assemblages for the area north of the Boconó Fault (i.e., *Watznaueria barnesae*, *Lithastrinus floralis*, *Tranolithus orionatus*, *Cretarhabdus* “*angustiforatus*”/*C. crenulatus*, *Broinsonia enormis*, and *Eiffelithus turriseiffeli*). However, they note that diagenesis may have influenced their determinations as it has caused pervasive recrystallization of calcite, and thus should be deemed “suggestive” ([7]). Ammonites, including *Acanthoceras*, *Sharpeiceras*, *Turrilites*, *Anisoceras*, and *Metoicoceras*, were all used to suggest a Cenomanian age for the La Aguada Member ([11, 12]). Additionally, Core ALP-6 from the western Maracaibo Basin established a Cenomanian age for the La Aguada Member based on planktonic foraminiferal biozones ([8], fig. 3 and references therein). Martinez and Hernandez [13] for the Molino River section (northern Colombia) and Ford and Houbolt [14]) for western Venezuela assumed an early Cenomanian age for the base of the La Luna Formation based on planktonic foraminifera specifically, *Globotruncana* (*Rotalipora*) aff. *montsalvensis* and “*Rotalipora* (*Thalmanninella*) *appeninica*”. Although the former taxon is indicative of the Cenomanian age ([15]), the latter may be latest Albian-Cenomanian (~101.9-94.03 Ma) according to [16]. Veiga and Dzelalijal [17] and Spickert [18] consider the La Luna Formation of Colombia on unknown grounds as Cenomanian-Santonian. Macsotay et al. [19] proposes assignment to mid-upper Cenomanian for the base of the La Luna Formation in Perijá and upper Albian?-lower Cenomanian more towards Lake Maracaibo (figure 2 in [19]). Most relevant here, perhaps, is the dating of the La Peña/San Felipe Sections by [20], located in the eastern part of the Maracaibo Basin on the eastern edge of the village of Chejendé, Trujillo, which is less than 10 km from the “Cementos Andinos” quarry. Despite their low diversity, nannofossils are common in the majority of the samples and the presence of *Eiffellithus turriseiffelii*, *Microstaurus chiastius*, and *Braarudosphaera* *africana*, and absence of *Gartnerago obliquum* indicate assignment of the base of the La Aguada Member in this section to the *Eiffellithus turriseiffelii-Gartnerago obliquum* zones. This would mean that the La Aguada Member was deposited no earlier than in the latest Albian to middle Cenomanian interval ([20]). However, only the base of the La Aguada Member was exposed, thus strongly suggesting an earliest Cenomanian age for MCNC-1830 that was recovered much higher in the section ([20], p. 352 and fig. 3A). Despite the debate on the exact age of the base of the La Luna Formation east of Lake Maracaibo (e.g., La Aguada Member), it seems that, based on the ammonite record, it becomes progressively younger westward ([11, 12, 19]).

**Extended environmental information on the La Luna Formation**

The high diversity in opportunistic predators within the La Luna Formation is reminiscent of the (shallower) ‘middle’ Cretaceous Western Interior Seaway environments with a high diversity of large marine vertebrates and thus be indicative of an abundance of food resources ([21]). Other parts of the La Luna sea, including the area around the Boconó Fault, have been suggested to have had generally nutrient-poor or “stressed” surface water conditions with periodic planktonic algal blooms and phases of replenishment of nutrients by upwelling or continental runoff ([7]).

The infrequent presence of microslumps and shell accumulations seem to indicate a water depth that approaches storm wave base in the area around the Boconó Fault ([7]). These anoxic conditions may have been present in the slope zone prior to the late Albian-early Cenomanian transgression ([21-23]). These anoxic conditions may have extended far up into the water column for extended periods of time as planktonic fauna and flora are little diversified or present as dwarf specimens (e.g. “flobigerinid” and “heterohelicid” foraminifera), the presence of the foraminifera *Heterohelix* and *Lunatriella* are indicative of stressed conditions, and for example the rarity of ammonites in the La Luna Formation around the Boconó Fault ([7]). Periodic oxygenation of the seafloor may have occurred as indicated by sparse occurrences of benthic foraminifera and *Inoceramus*; however, the presence of these organisms may also simply attest to their tolerance to anoxic conditions ([21]). Regardless, more work is needed to improve the scarce record of the Cretaceous vertebrates of Venezuela and to understand the complex ecological network of the La Luna Sea. This new plesiosaur is another indicator of the potential and abundance of marine vertebrates from the Cretaceous of Venezuela.

**References**

1. Garrity, C.P., Hackley, P.C. & Urbani, P.F. Digital shaded-relief map of Venezuela (Version 1.0). [Reston, Va]. *U.S. Geological Survey* (2004).
2. Garrity, C.P., Hackley, P.C. & Urbani, P.F. Digital shaded-relief map of Venezuela (Version 2.0). [Reston, Va]. *U.S. Geological Survey* (2009).
3. Garner, A. H. Suggested nomenclatural and correlation on geological formations in Venezuela. In: *Transactions of the American Institute of Mining and Metallurgy Engineers*, 677–684 (1926).
4. Bralower, T. J. & Lorente, M. A. Paleogeography and stratigraphy of the La Luna Formation and related Cretaceous anoxic depositional systems. *Palaios* **18 (**4-5), 301-304 (2003).
5. Hedberg, H. & Sass, L. Synopsis of the geologic formations of the western part of the Maracaibo Basin, Venezuela. *Boletín de Geología y Minería* **2-4**, 71–112 (1937).
6. Gonzalez de Juana, C., Iturrilde de Arocena, J. & Picard, X. *Geologia de Venezuela y de sus Cuencas Petroliferas. Foninves, Caracas*, **103**, 1 pp. (1980).
7. Tribovillard, N.P. *et al.* Cretaceous black shales of Venezuelan Andes: preliminary results on stratigraphy and paleoenvironmental interpretations. *Palaeogeography, Palaeoclimatology, Palaeoecology* **81**, 313-321 (1991).
8. Davis, C., Pratt, L., & Sliter, W. Factors influencing organic carbon and trace metal accumulation in the Upper Cretaceous La Luna Formation of the western Maracaibo Basin, Venezuela: in Barrera, E., and Johnson, C.C., eds., Evolution of the Cretaceous Ocean-Climate System. *Geological Society of America Special Paper* **332**, Geological Society of America, Boulder, 203–231 (1999).
9. Renz, O. Estratigrafía del Cretáceo en Venezuela occidental. *Boletín de Geología,* **5**(10), 3-48 (1959).
10. Renz, O. Die Ammonoidea im Stratotyp des Vraconien bei Sainte Croix (Kanton Waadt). *Schweizerische palaontologische Abhandlungen* **87**, 1-97 (1968).
11. Renz, O. The Cretaceous ammonites of Venezuela. Basel: Birkhäuser Verlag (1982).
12. Moody, J. M, & Maisey, J. G. New Cretaceous marine vertebrate assemblages from North-Western Venezuela and their significance. *Journal of Vertebrate Paleontology* **14**(1), 1-8 (1994).
13. Martinez, J. I., & Hernandez, R. Evolution and drowning of the Late Cretaceous Venezuelan carbonate platform. *Journal of South American Earth Sciences* **5**(2), 197-210 (1992).
14. Ford, A. B., & Houbolt, J. J. H. C. *The microfacies of the Cretaceous of western Venezuela* (Vol. 6). EJ Brill (1963).
15. Caron, M., & Spezzaferri, S. Scanning electron microscope documentation of the lost holotypes of Mornod, 1949: *Thalmanninella reicheli* and *Rotalipora montsalvensis*. *The Journal of Foraminiferal Research* **36**(4), 374-378 (2006).
16. Albino, A. M., Carrillo-Briceño, J. D. & Neenan, J. M. An enigmatic snake from the Cenomanian of Northern South America. *PeerJ* **4**, e2027. https://doi.org/10.7717/peerj.2027 (2016).
17. Veigal, R., & Dzelalija, F. A regional overview of the La Luna Formation and the Villeta Groups as Shale gas/Shale Oil in the Catatumbo Magdalena Valley and Eastern Cordillera Regions, Colombia. *American Association of Petroleum Geologists,* Search and Discovery Article #10565, 21 pp. (2014).
18. Spickert, A. Petroleum system analysis: Middle Magdalena Valley basin, Colombia, South America. MSC report. *University of Washington*, 1-48 (2014).
19. Macsotay, O., Erlich, R. N., & Peraza, T. Sedimentary Structures of the La Luna, Navay and Querecual Formations, Upper Cretaceous of Venezuela. *Palaios* **18**(4-5), 334-348 (2003).
20. De Romero, L. M. *et al.* An integrated calcareous microfossil biostratigraphic and carbon-isotope stratigraphic framework for the La Luna Formation, western Venezuela. *Palaios* **18**(4-5), 349-366 (2003).
21. Guinot, G., & Carrillo-Briceño, J. D. Lamniform sharks from the Cenomanian (Upper Cretaceous) of Venezuela. *Cretaceous Research* **82**, 1-20 (2018).
22. Erlich, R. N., Macsotay, O., Nederbragt, A. J., & Lorente, M. A. Palaeoceanography, palaeoecology, and depositional environments of Upper Cretaceous rocks of western Venezuela. *Palaeogeography, Palaeoclimatology, Palaeoecology* **153**, 203-238 (1999).
23. Mendez, C. E. La Formacion La Luna. Caracteristica de una cuenca anoxica en una plataforma de aguas someras. *Proceedings of the 7th Congreso Geologico Venezolano*, 852-866 (1981).
24. Carrillo-Briceño, J. D. & Sánchez-Villagra, M. R. In: Trebbau, P. & Pritchard, P. C. H. Tortugas del Pasado: una mirada al registro fósil en Venezuela. *Venezuela y sus tortugas.* (Eds Todtmann, O.), Caracas, Venezuela (2016).
25. Kellner, A. W. & Moody, J. M. Pterosaur (Pteranodontoidea, Pterodactyloidea) scapulocoracoid from the early Cretaceous of Venezuela. *Geological Society, London, Special Publications* **217**(1), 73-77 (2003).
26. Moody, J. M. First report of ichthyosaurian remains from the Cretaceous of Venezuela: *Anartia* **3**, p. 1-10 (1993).
27. Moody, J. M. Another Ichthyosaur. *The Fossil Record*, Dallas Paleontological Society, **Vol** **11** (4), 2-3 (1995).
28. Moody, J. M. Views of the past: the paleontology section of MBLUZ. *Revista Shell*, II Etapa, **2**(3),55-60 (1999).
29. Maisey, J. G. & Moody, J. M. A review of the problematic extinct teleost fish *Araripichthys,* with a description of a new species from the Lower Cretaceous of Venezuela. *American Museum Novitates* **3324**, 1-27 (2001).
30. Sánchez-Villagra, M. R., Brinkmann, W. & Lozsán, R. The Paleozoic and Mesozoic vertebrate record of Venezuela: An overview, summary of previous discoveries and report of a mosasaur from the La Luna Formation (Cretaceous). *Paläontologische Zeitschrift* **82**(2), 113-124 (2008).
31. Odreman Rivas, O. E. & Medina, C.J. Vertebrados fosiles de Venezuela, seeuencia, relaciones con otros paises de America del Sur. - *Cuadernos de Geologia, Ministerio de Energia y Minas* **1**, 60-86 (1984).
32. MINISTERIO DE ENERGIA Y MINAS. Lexico Estratigraifico de Venezuela, 3 'd ed. - *Boletin de Geologia* **12**, 1-828 (1997).
33. Carrillo-Briceño, J. D., Ayala, R., Chávez-Aponte, E. O. & González-Barba, G. Registro de *Serratolamna serrata* (Elasmobranchii: Serratolamnidae) en el Cretácico superior (Maestrichtiense) de los Andes Venezolanos [Record of *Serratolamna serrata* (Elasmobranchii: Serratolamnidae) from the upper Cretaceous (Maastrichtian) of Venezuelan Andes]. *Geominas* **36**(47), 160-163 (2008).
34. Weiler, W. Fischreste aus der Umgebung von San Cristobal, SW. Venezuela. *Zentralblatt für Mineralogie, Geologie und Paläontologie* (B) v. **1949**, p. 240–255 (1940).
35. Mutter, R. J., Iturralde-Vinent, M. & Carmona, J. F. The first Mesozoic Caribbean shark is from the Turonian of Cuba: *Ptychodus cyclodontis* sp. nov. (?Neoselachii). *Journal of Vertebrate Paleontology* **25**, 976–978. https//:doi:10.1671/02724634 025[0976:TFMCSI]2.0.CO;2 (2005).
36. Carrillo-Briceño, J. D. Presencia del genero “*Ptychodus*” (Elasmobrachii: Ptychodontidae) en el Cretacico superior de los Andes de Trujillo, Venezuela. *Geominas* **37**, 207–210 (2009).
37. Carrillo-Briceño, J. D. Presencia de *Ptychodus mortoni* (Elasmobranchii: Ptychodontidae) en el Cretacico superior de Venezuela. *Revista Geológica de América Central* **46**, 145-150 (2012).
38. Carrillo Briceño, J. D., Alvarado-Ortega, J. & Patiño Torres, C. Primer registro de *Xiphactinus* Leidy, 1870 (Teleostei, Ichthyodectiformes) en el Cretácico Superior de América del Sur (Formación La Luna, Venezuela). *Revista Brasileira de Paleontologia*. **15**(3), 327-35 (2012).
39. Liddle, R. A. Geology of Venezuela and Trinidad. J. P. Macgowan, Fort Worth, Texas, 552 pp (1928).
40. Rod, E. & Mayne, W. Revision of Lower Cretaceous stratigraphy of Venezuela. *Bulletin of the American Association of Petroleum Geologists* **38**, 193-283 (1954).
41. Albino, A. M., Rothschild, B., Carrillo-Briceño, J. D., & Neenan, J. M. Spondyloarthropathy in vertebrae of the aquatic Cretaceous snake *Lunaophis aquaticus*, and its first recognition in modern snakes. *The Science of Nature* **105**(9), p. 51 (2018).
42. Carrillo-Briceño, J. D. & Lucas, S. G. The first tooth set of *Ptychodus atcoensis* (Elasmobranchii: Ptychodontidae), from the Cretaceous of Venezuela. *Swiss Journal of Palaeontology* **132**(1), 69-75 (2013).
43. Sachs, S. Dinosaurier in Venezuela. *Dinosaurier Magazin*, **Heft ¾** (1991).
44. Barrett, P. M. *et al.* A palaeoequatorial ornithischian and new constraints on early dinosaur diversification. *Proceedings of the Royal Society B: Biological Sciences* **281**(1791), 20141147 (2014).
45. Russell, D. C.; Odreman Rivas, O.; Battail, B. & Russell, D.A. Découverte de vertébrés fossils dans la Formation de La Quinta, Jurassique du Vénézuela Occidental. - *Comptes Rendus de l'Académie des Sciences*, Paris, Série II **314**, 1247-1252 (1992).
46. Sánchez-Villagra, M. R. & Clark, J. M. An ornithischian from the Jurassic of the Venezuelan Andes. *Journal of Vertebrate Paleontology* **14** (Suppl. 3), 44A (1994).
47. Kunding, E. Las rocas precretáceas de los Andes centrales de Venezuela con algunas observaciones sobre su tectónica. *Boletín de Geologfía y Minas*, Caracas **2** (2 & 4), 21-43 (1938).
48. Moody, J. M. Theropod teeth from the Jurassic of Venezuela. *Boletín de la Sociedad Venezolana de Geología* **22**, 37-42 (1997).
49. Langer, M. C., Rincón, A. D., Ramezani, J., Solórzano, A., & Rauhut, O. W. New dinosaur (Theropoda, stem-Averostra) from the earliest Jurassic of the La Quinta formation, Venezuelan Andes. *Royal Society Open Science* **1**(2), 140184 (2014).
50. Bowen, J. M. Estratigraffa del precretácico en la parte norte de la Sierra de Perijá. - Congreso Geologico Venezolano, *Memorias* **2**, 729-761 (1972).
51. Benedetto, G. & Odreman Rivas, O. Nuevas evidencias paleottologicas en la Formacion La Quinta, su edad y correlacion con las unidades aflorantes en la Sierra de Perijá y Cordillera oriental de Colombia. V. Congreso Geologico Venezolano, *Memorias* **1**, 87-106 (1977).
52. Pierce, G. R. Geología de la Cuenca de Barinas. - III. Congreso Geológico Venezolano, Caracas, 1959, *Memorias* **1**, 214-276 (1960).
53. Sánchez, T. M. & Lorente, M. A. Paleoambiente del Miembro Quevedo (Formación Navay) en las proximidades de Santa Bárbara. - V. Congreso Geológico Venezolano, *Memorias* **1**, 107-133 (1977).
54. Pierce, G. R. & Wells, S. P. Primer hallazgo de restos de mosasauro en el Cretáceo de Santa Bárbara de Barinas, Venezuela. - Sociedad Venezolana de Ciencias Naturales, *Boletin* **17**, 21-24 (1956).
55. Macsotay, O., Vivas, W., Pimentel, N. & Bellizia, A. Estratigrafia y tectónica del Cretáceo-Paleoceno de las islas al norte de Puerto La Cruz-Santa Fé y regiones adyacentes. Excursión. - VI. Congreso de Geología de Venezuela, Caracas, *Memorias* **10**, 7125-7175 (1985).
56. Colbert, E. A new Cretaceous plesiosaur from Venezuela. *American Museum Novitates* **1420**, 1-22 (1949).
57. Jaillard, E., Cordova, A., Mazin, J.-M. & Mourier, T. La transgression du Cénomanien supérieur-Turonien inférieur dans le région de Jaén (Nord du Pérou): donnés sédimentologique et stratigraphiques: découverte du premier saurien marin du Pérou. *C. R. Acad. Sc. Paris*, t. **301**, Série II, no. 20**,** 1429-1432 (1985).
58. Odreman Rivas, O. E. & Benedetto, G. Paleontología y Edad de la Formación Tinacoa, Sierra de Perijá, Estado Zulia, Venezuela. V Congreso Geológico Venezolano, *Memorias* **1**, 15-32 (1977).
59. Brito, P. M., Janvier, P. A ptychodonid (Chondrichthyes, Elasmobranchii) from the Upper Cretaceous of South America. *Geodiversitas* **24**, 785–790 (2002).
